# Post-intubation tracheal stenosis after management of complicated aortic dissection: a case series

**DOI:** 10.1186/s13019-015-0357-z

**Published:** 2015-11-04

**Authors:** Jia Liu, Chun-Peng Zhang, Ye Li, Su Dong

**Affiliations:** 1 Department of Thyroid Surgery, First hospital of Jilin University, Changchun, Jilin China; 2 Department of Cardiovascular Surgery, First hospital of Jilin University, Changchun, Jilin China; 3 Department of Radiology, First hospital of Jilin University, Changchun, Jilin China; 4 Department of Anesthesia, First hospital of Jilin University, No.71st Xinmin ST, Changchun, Jilin 130021 China

**Keywords:** Tracheal intubation, Tracheal stenosis, Aortic dissection

## Abstract

**Background:**

Patients undergoing total aortic arch replacement or aortic dissecting aneurysmectomy are generally managed with medications to control hypotension and blood coagulation to minimize mortality and morbidity. However, prolonged mechanical ventilation via tracheal intubation increases the risk of tracheal stenosis in such patients.

**Case presentation:**

We present 2 cases (a 49-year-old woman and a 62-year-old man) of post-intubation tracheal stenosis occurring after surgery for the correction of complicated aortic dissection; both cases were successfully managed by tracheal cryotherapy.

**Conclusion:**

Continuous monitoring of cuff pressure and regular cuff palpation are necessary to minimize the incidence of tracheal stenosis. If the patients have concomitant local or systemic infection, adequate preventive measures should be taken to reduce the incidence of post-intubation tracheal stenosis. Tracheal cryotherapy is recommendable for the management of post-intubation tracheal stenosis.

## Background

Total aortic arch replacement and aortic aneurysmectomy are frequently performed at large medical centers for the management of patients with acute aortic dissection. Generally, patients with complicated aortic arch lesions also have systemic vascular comorbidities. Patients undergoing total aortic arch replacement or aortic aneurysmectomy require special care for the intraoperative management of blood pressure and blood coagulation during the surgery in order to minimize morbidity and mortality associated with the procedure; however, prolonged mechanical ventilation during tracheal intubation increases the risk of tracheal stenosis [1, 2]. However, reports on the management of tracheal stenosis in patients undergoing surgery for aortic arch disease are limited. In this paper, we present two cases of tracheal stenosis caused by prolonged mechanical ventilation in patients who underwent surgery for the treatment of complicated aortic dissection.

### Case presentation

#### Case 1

**Fig. 1:**
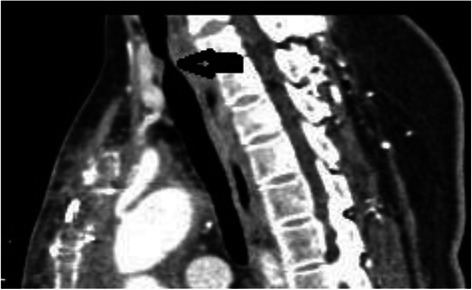
Tracheal stenosis in Case 1: A computed tomographic scan image of a 49-year-old woman (*case 1*) showing severe tracheal stenosis, with diameter of 3.5 mm for a length of over 1 cm near the region of the thyroid isthmus (*arrow*)

The patient was a 49-year-old woman presenting to the emergency department of our institution with severe chest pain; the patient was diagnosed with acute aortic dissection (Standford type A) and referred to the Department of Cardiovascular Surgery. Twenty-four hours after the referral, the patient underwent emergent total aortic arch replacement and distal repair with the stented elephant trunk procedure. After the procedure, the patient was transferred to the intensive care unit for the further management of mechanical ventilation. To prevent postoperative bleeding, the arterial blood pressure was maintained at 90/60 mmHg. Seventy-two hours after the surgery, the patient was extubated, and the patient discharged was discharged 12 days later. Six months after the operation, the patient was admitted for the management of recurrent episodes of laryngeal stridor and dyspnea, which had necessitated multiple hospital admissions after the surgery. Arterial blood gas analysis on admission showed that the patient had mild hypoxemia (PaO2: 56 mmHg). A computed tomography scan was performed, which revealed that the patient had tracheal narrowing (diameter of the 3.5 mm over a length of 1 cm near the region of the thyroid isthmus (Fig. 1). The patient then underwent tracheal cryotherapy. Five days after the procedure, no signs of upper airway obstruction were evident, and the patient was discharged.

#### Case 2

**Fig. 2:**
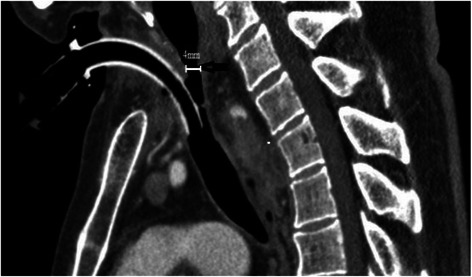
Tracheal stenosis in Case 2: A computed tomographic scan image of a 62-year-old man (*case 2*) showing severe tracheal stenosis with diameter of 4 mm above the site of tracheostomy (*arrow*)

A 62-year-old man was admitted to the emergency department with severe dyspnea since 2 h, along with cough and expectoration and generalized paresthesia. On admission, the patient was in extremis and had tachypnea (respiratory rate, >35 cycles/min). Arterial blood gas analysis showed that the patient had severe hypercarbia (pHa, 7.23; PaCO2, 76 mmHg) and hypoxemia (PaO2, 16 mmHg). History taking revealed that the patient had undergone aortic dissecting aneurysmectomy 2 months before admission, after which the patient had been on ventilation support for 6 days. Therefore, the patient was immediately transferred to the intensive care unit of the Department of Cardiovascular Surgery. Shortly after the transfer, the patient was rendered unconscious and developed apnea. Immediately, endotracheal intubation was performed. Laryngoscopy was performed, and the glottis was visualized to be normal; despite this, the passage of a tracheal tube (internal diameter, 7.5 mm) was not possible deeper than 2 cm beyond the vocal cords because of firm resistance. Similarly, it was difficult to pass even thinner tracheal tubes (internal diameters, 5.0–7.0 mm) or a flexible gum elastic bougie beyond the obstruction. Therefore, ventilator support was established with a bag and mask and laryngeal mask. When the patient regained consciousness, he was writhing and required sedation. Surgical consultation was immediately sought, and tracheostomy was considered. Tracheal stenosis was strongly suspected; however, the location of the stenosis was unknown although it was believed to be below the level of surgical tracheostomy. A pediatric tracheal tube (internal diameter, 4.5 mm) was then advanced through the trachea until resistance was encountered again. Therefore, the location of the resistance was believed to be above the tracheostomy, and surgical correction of the stenosis was promptly encountered. Once emergency tracheotomy was performed, ventilation could be established without the need for excessive high airway pressure, and the wheezing sound on auscultation of the chest was no longer present. A computed tomography scan image showed that the trachea had narrowed to a diameter of 4 mm above the site of tracheostomy (Fig. 2). Subsequently, the patient underwent tracheal cryotherapy and was discharged from the hospital 15 days later.

## Discussion

Aortic dissections and aneurysms are rare conditions associated with a high risk of deterioration during prehospital care. Emergency surgery is necessary in both these conditions to prevent the rupture of the lesions. Nevertheless, the mortality and morbidity associated with the required surgical procedures are higher than those noted for other cardiac surgeries, such as valvular surgery and coronary artery surgery, mainly because of the requirement of tracheal intubation and postoperative ventilation support for more than 48 h. Control of hypotension and sedation are required in the intensive care unit after such cardiovascular surgeries. Control of hypotension-induced tracheal mucosal hypoperfusion is an important risk factor for tracheal stenosis. However, limited reports have been published thus far on the relationship between aortic arch disease and postoperative tracheal stenosis.

Tracheal stenosis secondary to endotracheal intubation may be caused by trauma, infection, or iatrogenic airway injury [3]. Generally, the oral endotracheal tube is used to provide mechanical ventilator support while the patient is under general anesthesia and during the postoperative management. However, despite advances in the diagnostic and therapeutic techniques and patient care in the intensive care unit, the incidence of tracheal stenosis at the cuff of the tube continues to remain high [4]. Stenosis has been reported to be mainly caused by the pressure exerted by the cuff on the tracheal mucosa. At a cuff pressure of >30 mmHg, there is an increase in mucosal capillary perfusion pressure, which leads to mucosal ischemia and consequent inflammation of the tracheal cartilages. These pathological changes may eventually lead to fibrosis of circumferential lesions, resulting in progressive tracheal stenosis [5]. Ischemic injury can occur even 15 min after insufflation of the cuff, and subsequent fibrotic changes within the following 3–6 weeks. Although the use of large-volume, low-pressure cuffs markedly reduces the occurrence of cuff injury, tracheal stenosis continues to occur at a high frequency, with the incidence of post-intubation tracheal stenosis in intensive care units being 6–21 %, although only 1–2 % of patients are symptomatic or have severe stenosis [6]. The diagnosis of tracheal stenosis is often missed, and the related symptoms are generally evident only when stenosis of 30 % of the original diameter of the trachea has occurred. In fact, the diagnosis may be delayed for as long as three months after the intubation [7]. Several risk factors of post-intubation tracheal stenosis have been recognized thus far, including the size of the endotracheal tube relative to the tracheal lumen, frequent replacement of the endotracheal tube, traumatic intubation, concurrent infection, blood pressure during the intubation period, female gender, estrogen effect, steroid administration, obesity, smoking history, etc. [8, 9].

In this paper, we presented two cases of post-intubation tracheal stenosis occurring secondary to surgical procedures for the management of aortic dissection with complications. The comorbidities complicating the aortic dissection in the two cases were as follows: hypotension, prolonged ventilation, and possible systemic vascular disease. To avoid postoperative aortic anastomotic bleeding, blood pressure is strictly monitored and controlled and anticoagulants are regularly used, which may result in hypoperfusion of the tracheal mucosa. Additionally, the cuff pressure is not measured as part of the routine monitoring, whereby ischemic injury of the tracheal mucosa cannot be eliminated in all cases. Both the patients in this study had been on postoperative ventilation support for a long duration—one for 72 h and the other for 6 days. This period was longer in the second case because the patient had the additional complications of atelectasis and systemic infection. Further, the patient in Case 2 also had more severe and emergent symptoms, which necessitated tracheostomy. The duration between discharge and re-hospitalization for dyspnea in Cases 1 and 2 was 6 months and 2 months, respectively; this suggests that longer duration of ventilation support and infection probably results in a more rapid onset of tracheal stenosis.

Non-specific presentation and normal examination finding often result in tracheal obstruction being overlooked as a diagnosis until patients present acutely. Once the tracheal stenosis is diagnosed, surgical options should be considered. Long term tracheostomy may become necessary for patients with complex tracheal disease. Non-resectable tracheal stenosis can be successfully managed by interventional bronchoscopy, with therapeutic options including airway dilation, local tissue destruction and airway stenting, laser and cryotherapy [10]. However, Bisson et al. [11] reported that the risk of expansion restenosis is about 90 % if the procedure is limited to a single dilation. In the event of restenosis, further medical treatment or even surgical management with tracheal resection or reconstruction may be required. Tracheal cryotherapy has been reported to cure benign tracheal neoplasm with good tolerance and favourable prognosis in children [12]. In our patients, tracheal cryotherapy yielded satisfactory outcomes and merits further examination for widespread application. Successful methods for the treatment of tracheal stenosis are yet to be developed. It is reported that the suppression of c-Myc expression represents a potential strategy for the treatment of tracheal stenosis. The airway-targeted gene therapy can be developed as a safe and effective therapeutic agent for the treatment of airway stenosis [13].

## Conclusion

In summary, we presented two cases of tracheal stenosis occurring after the surgical procedures for the repair of aortic dissection with complications; such cases of aortic dissection surgery are often associated with prolonged tracheal intubation for mechanical ventilation and possibility of comorbidity with systemic vascular lesions and hypotension. Continuous monitoring of cuff pressure and regular cuff palpation are necessary to minimize the incidence of tracheal stenosis. If the patients have concomitant local or systemic infection, adequate preventive measures should be taken by the anesthesiologists and surgeons to reduce the incidence of post-intubation tracheal stenosis. Our experience in these two cases suggests that tracheal cryotherapy is recommendable for the management of post-intubation tracheal stenosis.

## Consent

Written informed consent was obtained from the patient for publication of this case report and accompanying images. A copy of the written consent is available for review by the Editor-in-Chief of this journal.
